# Macular Blood Flow and Pattern Electroretinogram in Normal Tension Glaucoma

**DOI:** 10.3390/jcm11071790

**Published:** 2022-03-24

**Authors:** Soo Ji Jeon, Kyoung In Jung, Chan Kee Park, Hae-Young Lopilly Park

**Affiliations:** 1 Department of Ophthalmology, Bucheon St. Mary’s Hospital, College of Medicine, The Catholic University of Korea, Bucheon 14647, Korea; sj8801@gmail.com; 2 Department of Ophthalmology, Seoul St. Mary’s Hospital, College of Medicine, The Catholic University of Korea, Seoul 06591, Korea; ezilean@hanmail.net (K.I.J.); ckpark@catholic.ac.kr (C.K.P.)

**Keywords:** normal tension glaucoma, pattern electroretinogram, optical coherence tomography angiography, macular vessel density

## Abstract

Purpose: To investigate whether macular vessel density (VD) was associated with the pattern electroretinogram (PERG) in normal tension glaucoma (NTG). Design: Cross-sectional study. Methods: Seventy-six eyes from patients with NTG were included in this study. Macular VD was calculated from the superficial retinal layer, including the retinal nerve fiber layer (RNFL) and ganglion cell layer (GCL), using the built-in software provided with the optical coherence tomography angiography (OCTA) device. Functional parameters were obtained from standard automated perimetry (SAP) and PERG, using a commercial ERG stimulator. Moreover, structural parameters, such as peripapillary RNFL and macular ganglion cell/inner plexiform layer (GCIPL) thickness, were measured using OCT. Results: Patients with higher VD had higher N95 amplitude (*p* = 0.048). Macular VD was significantly correlated with N95 amplitude, irrespective of disease severity (r = 0.352, *p* = 0.002 for the total subjects and r = 0.276, *p* = 0.043 for mild glaucoma). According to regression analyses, N95 amplitude and macular VD were bidirectional significant factors (*p* = 0.035 and 0.019, respectively). For patients with mild to moderate glaucoma, N95 amplitude and macular VD were also significantly associated bidirectionally, according to regression analyses (*p* = 0.032 and 0.040, respectively). Conclusions: Macular VD was significantly associated with N95 amplitude from PERG. The correlation was prominent in early glaucoma, in contrast to the other structural or functional parameters. When considering that PERG represents the objective function of the retinal ganglion cell (RGC), macular VD was associated with RGC dysfunction before the functional change became apparent on SAP.

## 1. Introduction

Glaucoma is a disease characterized by morphological changes in the optic disc and typical visual field (VF) defects of the corresponding topographic area [1]. Therefore, standard automated perimetry (SAP) is a key testing modality that can evaluate visual function in glaucoma [2]. However, SAP has real-life limitations when used for the clinical assessment of patients. In early glaucoma, the discrepancies in the detection of retinal nerve fiber layer (RNFL) loss and alteration of visual function are quite substantial [3,4,5]. One study reported that SAP could not detect VF abnormalities until 25–35% of the retinal ganglion cells (RGC) were lost to cell death [6]. Moreover, SAP depends on the patients’ subjective response and may not provide a completely objective representation of the function of the RGC. External factors, such as media opacity, psychologic effects, or other environmental events at the time of test could produce different results [7,8,9].

Pattern electroretinogram (PERG) has been a long-proposed method for evaluating visual function to overcome the limitations of the VF test [10]. RGC function in glaucoma could be estimated directly and objectively via PERG [11]. The usefulness of PERG has been emphasized, particularly, for detecting the early stage of the disease [12,13,14]. The previous studies conducted by our group suggested that the amplitude of the PERG wave decreased in preperimetric glaucoma [15], and it was associated with the optic disc morphology in suspected glaucoma with increased cup-disc ratio (CDR), before functional loss was detected by SAP [16].

Optical coherence tomography angiography (OCTA) is a method that could visualize the blood flow of the retina. Several studies reported about the vessel density (VD) changes in the peripapillary area and macula in patients with glaucoma, and concurrently found that VD decreased with the increase in the severity of glaucoma [17,18,19,20]. However, there is controversy regarding whether the decrease in VD is caused by RNFL loss or if VD is an independent marker of visual function of glaucoma. One possibility is that VD may represent the metabolic status of the RGC and the decreased metabolic demand resulting from RGC dysfunction could be detected as reduced VD in glaucoma. Therefore, we hypothesized that RGC dysfunction could be detected in the early stage using PERG, before the changes became apparent on SAP, and this PERG result could have correlation with the VD of OCTA.

The purpose of this study was to investigate the association between PERG parameters and VD obtained from OCTA in glaucoma patients. If VD from OCTA correlates with PERG, which is an objective electrophysiologic test, we aimed to identify the possibility of clinical application of OCTA, as an additional parameter of visual function.

## 2. Materials and Methods

### 2.1. Subjects

This study was conducted according to the tenets of the Declaration of Helsinki and was approved by the Institutional Review and Ethics Boards of Seoul St. Mary’s Hospital, South Korea. A total of 102 eyes of patients with normal-tension glaucoma (NTG) from the glaucoma clinic of Seoul St. Mary’s Hospital between July 2018 and December 2020 were included in this study. The need for written informed consent was waived by our Review Board.

Patients were determined to have glaucomatous VF defects if they satisfied the following conditions: a group of 3 or more spots, 2 of the points had a chance of presence <5% in normal subjects, and 1 spot had a chance of presence <1% in normal subjects for the pattern deviation plot. The VF results were considered reliable if the fixation loss was <20%, false-positive rate was <15%, and false-negative rate was <15%. All subjects exhibited manifestations of glaucomatous optic discs such as increased CDR with localized thinning of the neuroretinal rim, generalized loss of the disc rim, or RNFL defects corresponding to VF defects. Mild glaucoma was defined as mean deviation (MD) of Swedish interactive threshold algorithm (SITA) 24-2 ≥ −6 dB, moderate glaucoma was defined as −6 dB ≥ MD ≥ −12 dB, and severe glaucoma was defined as −12 dB ≥ MD according to Hodapp–Anderson–Parrish criteria [21]. All subjects had well-controlled disease status with IOP-lowering eyedrops. Two glaucoma specialists (SJJ and KIJ) reviewed the medical records to assess disease status and all patients had stable VF and OCT results for recent 3 years. In cases of disagreement between two specialists, the third glaucoma specialist (HYP) re-assessed the disease status. Even patients with stable disease, who underwent glaucoma laser or surgery were excluded.

The other inclusion criteria were as follows: (1) best-corrected visual acuity (BCVA) of 20/30 or better, (2) intraocular pressure (IOP) ≤ 21 mmHg without the use of IOP-lowering eyedrops at first visit to the clinic, (3) open angle on gonioscopy, and (4) spherical equivalent within ±5.0 diopters. When both eyes fulfilled the inclusion criteria, one eye per individual was randomly selected.

Patients were excluded if they had a history of uveitis; retinal diseases such as retinal vein obstruction, macular degeneration, and diabetic retinopathy; a history of intraocular surgery except for uncomplicated cataract extraction. Patients with any optic-nerve-related disease besides glaucoma, and/or a history of systemic or neurological diseases that might affect the VF or PERG were excluded.

All subjects underwent complete ophthalmic examinations including slit-lamp examination, Goldmann applanation tonometry, gonioscopy, and dilated fundus bimicroscopy. SAP 24-2 was performed using SITA program (Humphrey Visual Field Analyzer; Carl Zeiss Meditec, Inc., Dublin, CA, USA). Circumpapillary RNFL thickness and ganglion cell/inner plexiform layer (GCIPL) thickness were measured using Cirrus spectral-domain optical coherence tomography (SD-OCT; Carl Zeiss Meditec, Dublin, CA, USA). Only well-focused OCT images with signal strengths >6 were included.

### 2.2. Electroretinography

A commercial PERG system (Neuro-ERG, Neurosoft, Ivanovo, Russia) was used to record electroretinogram. One trained examiner performed the examinations. Two Ag/AgCl ground electrodes were placed on both earlobes, and reference electrodes were placed on the ipsilateral lower eyelid. Detailed description of the examination was provided in our previous study [15,16]. In short, the subjects with proper optical correction and undilated pupils were seated in front of a display. A checkerboard pattern with a mean luminance of 105 cd/m^2^ was reversed at the rate of 4 reversals per second at a 60-cm distance. The stimulus display covered 48 × 33° of the VF, with each check size of 1.8° visual angle. Subjects focused on the red fixation point at the center of display, and both eyes were examined simultaneously. The reproducibility of the ERG results was identified by previous studies using the intraclass correlation coefficient of the randomly selected measurements [15,16].

### 2.3. OCTA Imaging and VD Measurement

The macular VD was measured using a swept-source OCTA device (DRI OCT Triton; Topcon, Tokyo, Japan). The device used a wavelength of 1050 nm and scanning speed of 100,000 A-scans per second. A 4.5 × 4.5 mm macular scan was obtained in the “angio macula” mode, and the acquired image was automatically centered over the fovea. An active eye tracker system was used to minimize the motion artifacts on the image. The examinations were performed by one trained examiner and only images with quality scores over 70 were selected to ensure that the OCTA images would be of superior quality. The automatically segmented retinal vascular plexus consists of the superficial layer and deep layer. The VD of the superficial layer was used as the macular VD in this study; it was calculated automatically after applying the built-in software projections removal algorithm to the deep retinal and choriocapillary layers. The superficial vascular plexus extends from 2.6 μm below the internal limiting membrane to 15.6 μm below the junction of the inner plexiform layer (IPL) and inner nuclear layer (INL) (IPL/INL) according to the default settings of the device. The VD measurements were composed of five subfields using the Early Treatment Diabetic Retinopathy Study (ETDRS) grid overlay combining the two inner rings [22]. The average macular VD was calculated as the mean value of VD of the superior, nasal, inferior, and temporal areas. Participants were divided into the higher VD and lower VD groups based on the mean value of the average VD obtained from the total study population.

### 2.4. Statistical Analysis

All data are presented as the mean ± standard deviation. Student’s t test was used to compare the variables of the higher and lower VD groups. Linear regression analyses were conducted to evaluate the significant factors affecting macular VD or PERG N95 amplitude, and the analyses were applied to the total subjects or only to patients with mild to moderate glaucoma, respectively. All statistical analyses were performed using SPSS version 24.0 (SPSS Inc., Chicago, IL, USA). Fially, *p* < 0.05 was considered to be statistically significant.

## 3. Results

A total of 102 eyes of patients with NTG, who met the eligibility criteria, underwent VF, OCT, PERG and OCTA examinations. Of the 102 eyes, 17 eyes (16.7%) were excluded from further analysis, owing to the poor quality or presence of motion artifacts on the OCTA images and nine eyes were excluded (8.8%) because the results of SAP or PERG were unreliable. The remaining 76 eyes with NTG were included in the final analysis. Table 1 demonstrates the participants’ baseline characteristics. The IOP remained within the normal range throughout the study period in all participants. The MD distribution ranged from −0.01 to −31.47 dB, including mild, moderate and severe glaucoma patients. Among study subjects, the numbers of moderate and severe glaucoma patients were 10 and 12, respectively.

Participants were classified into the higher and lower VD groups based on the mean value of average macular VD, and their visual functional and structural parameters were compared (Table 2). N95 amplitude was higher in the higher VD group than in the lower VD group (5.52 μV vs. 4.65 μV, *p* = 0.048). Other parameters, such as RNFL thickness, GCIPL thickness, MD and PSD from SITA 24-2, P50 latency, N95 latency, and P50 amplitude, were not significantly different between the two groups.

**Figure 1:**
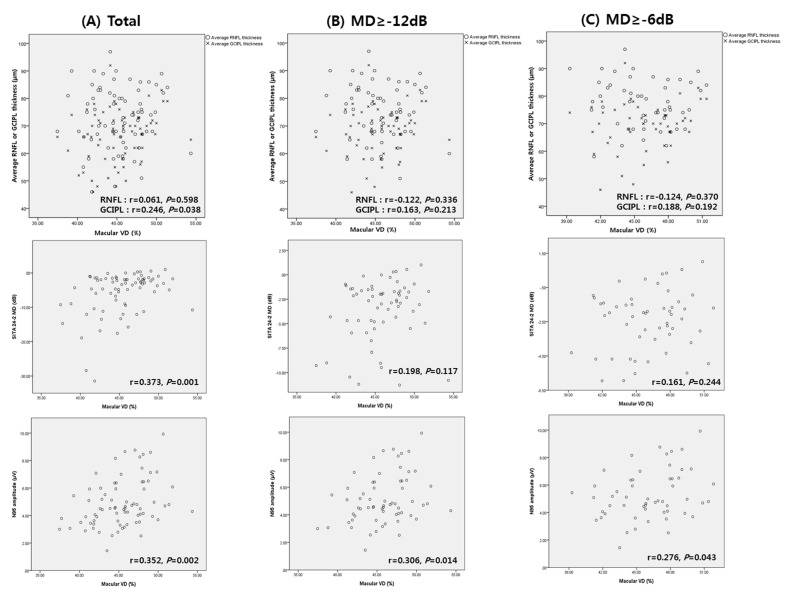
Scatter plots showing associations between macular vessel density (VD), average retinal nerve fiber layer (RNFL) thickness, average ganglion cell layer/inner plexiform layer (GCIPL) thickness, mean deviation (MD) of SITA 24-2, and N95 amplitude of pattern electroretinogram (PERG) from (**A**) total subjects, (**B**) mild to moderate glaucoma, and (**C**) mild glaucoma.

Scatter plots showing the correlations between macular VD, structural parameters (RNFL and GCIPL thickness), SITA 24-2, and PERG parameters were analyzed. Figure 1 demonstrates the association between macular VD and other parameters. In the total subjects, macular VD was correlated with GCIPL thickness (r = 0.246, *p* = 0.038), SITA 24-2 MD (r = 0.373, *p* = 0.001), and N95 amplitude (r = 0.352, *p* = 0.002; Figure 1A). However, in mild to moderate glaucoma or mild glaucoma patients, only N95 amplitude showed significant correlation with macular VD (r = 0.306, *p* = 0.014 for mild to moderate glaucoma; r = 0.276, *p* = 0.043 for mild glaucoma, Figure 1B,C). The MD of SITA 24-2 or GCIPL thickness were not significantly correlated with macular VD, in mild or mild to moderate glaucoma. RNFL thickness was not significantly associated with macular VD in any group.

**Figure 2:**
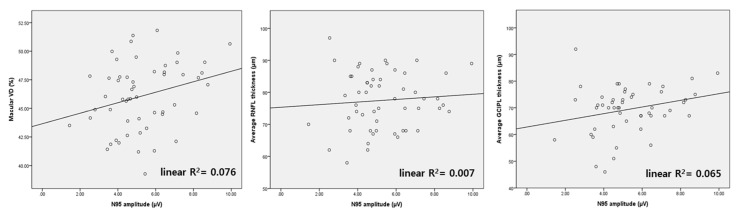
Scatter plots of N95 amplitude, macular vessel density (VD), and average RNFL thickness from mild glaucoma patients (MD ≥ −6 dB). The scatter plot of macular VD shows the most linear association with N95 amplitude among the three parameters.

Figure 2 shows the distribution of macular VD, RNFL and GCIPL thickness, according to N95 amplitude in mild glaucoma (MD ≥ −6 dB). Linear R^2^ was the lowest for RNFL thickness (linear R^2^ = 0.007). Linear R^2^ of macular VD was higher than that of GCIPL thickness (linear R^2^ = 0.076 and 0.065, respectively). In short, the association between N95 amplitude and macular VD showed the greatest linearity, compared to that of N95 with GCIPL or RNFL.

**Figure 3:**
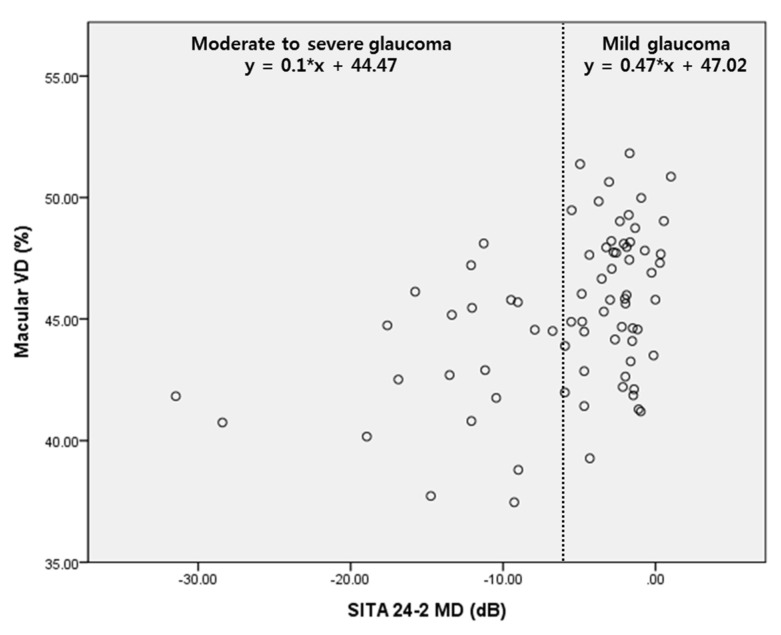
Scatter plot showing the relationship between MD of SITA 24-2 and macular vessel density (VD). There was more delicate change of macular VD according to MD in mild glaucoma.

According to Figure 3, the slope of the regression graph between SITA 24-2 MD and macular VD differed, depending on the severity of the glaucoma. The regression line was steeper for mild glaucoma than that for moderate to severe glaucoma. Hence, macular VD could represent the delicate distribution better than SITA 24-2 MD in patients with mild glaucoma.

Table 3 and Table 4 show the results of linear regression analyses, conducted to identify the variables associated with macular VD, based on the severity of the glaucoma. With total subjects, multivariate regression analysis with the total study subjects revealed that N95 amplitude was the only significant factor affecting macular VD (*p* = 0.035, Table 3). When performing subgroup analysis only in mild to moderate glaucoma patients (MD ≥ −12 dB), N95 amplitude was only associated with macular VD, according to several multivariate regression models (*p* = 0.032 for model 2 and 0.033 for model 3, Table 4). Contrary to our expectation, structural parameters, such as RNFL and GCIPL thickness, were not related with macular VD, in all multivariate analyses.

Table 5 and Table 6 present the results of linear regression analyses conducted to determine the factors associated with N95 amplitude, according to the severity of the glaucoma. With the total subjects, univariate analysis revealed that RNFL thickness, GCIPL thickness, and macular VD were significant factors in the total study subjects (*p* = 0.030 for RNFL thickness; 0.001 for GCIPL thickness; <0.001 for superior macular VD; 0.017 for temporal macular VD; 0.002 for average macular VD, Table 5). However, in multivariate analysis, only macular VD was significantly associated with N95 amplitude (*p* = 0.019, Table 5). For mild to moderate glaucoma, GCIPL thickness and macular VD were significantly associated with N95 amplitude in the univariate analysis (*p* = 0.020 for GCIPL thickness; 0.001 for superior macular VD; 0.046 for temporal macular VD; 0.014 for average macular VD, Table 6). Multivariate analysis, comprising both variables, revealed that GCIPL and average macular VD were still significantly associated with N95 amplitude (*p* = 0.042 and 0.040, respectively, Table 6).

## 4. Discussion

The present study demonstrated the association between PERG amplitude, macular VD and other various structural and functional parameters, including OCT and visual field test, in NTG patients. Macular VD was correlated with the N95 amplitude of PERG, which could reflect RGC function objectively. In subgroup analyses, the association between macular VD and N95 amplitude was remarkable in patients with mild glaucoma, while the other structural parameters did not exhibit any meaningful correlation. Further, N95 amplitude was consistently associated to macular VD, irrespective of disease severity, but was not related to RNFL thickness in mild glaucoma patients. This result could be interpreted as meaning that macular VD may be representative of RGC function as PERG, before the onset of structural (GCIPL or RNFL thickness) or functional (SAP) changes.

Studies have reported that several factors hinder the evaluation of visual function in early glaucoma using the VF test [23,24,25]. The occurrence of RGC dysfunction before cell death in early-stage disease could lead to different visual function compared to normal status, but the dysfunctional RGC could not be fully evaluated using SAP. Therefore, a substantial amount of RGC death precedes definite VF defects detected in SAP [6]. Several efforts were made to objectively assess visual function in early glaucoma using electrophysiological tests to overcome the limitations of SAP. Ventura et al. found alterations in the PERG after IOP reduction in patients with glaucoma, except for those with severely impaired visual function. This implies the existence of a dysfunctional, but viable, RGC in early glaucoma [26]. A study by Jafarzadehpour and associates reported the different PERG pattern of dysfunctional RGC before cell loss [14]. Another previous study suggested that the reduction in the electrical activity of RGC was disproportionately greater than the number of lost RGC in early glaucoma, indicating that the dysfunctional RGC could be identified using PERG [5].

Recently, several studies evaluated the possibility of using OCTA-derived VD as a visual function parameter. Yarmohammadi et al. reported that the association between OCTA and VF was stronger than that of other structural parameters obtained by OCT [27]. A study of patients with non-arteritic anterior ischemic optic neuropathy demonstrated that the vascular changes depicted by OCTA showed strong functional correlations with VF defects [28]. Moreover, a couple of studies suggested that macular VD changes were independent of structural changes in the macula. A longitudinal cohort study by Shoji et al. identified that macular VD could detect disease progression, even though the structure parameters remained unchanged [29]. Hou et al. recently reported that the reduction in macular VD was identifiable in preperimetric glaucoma and was even faster than GCIPL thinning [30]. Both studies reported that the reduction in VD was more detectable than structure thinning.

Therefore, we deduced that it would be worthwhile to identify whether OCTA was associated with visual function parameters, such as PERG, that represent objective RGC function. From the results of this study, objective RGC function, represented by PERG, was associated with macular VD, and it supported the possibility that macular VD could be a surrogate of visual function in early glaucoma. Dysfunctional neurons may have decreased oxygen demands, and also, inversely, metabolically challenged neurons could have depletion of function [31,32]. The RGC dysfunction and decreased blood supply, which transfer oxygen, essential for neuronal function, would be closely related. Macular VD was correlated with the electrophysiological results of RGC, independent of macular structure (i.e., GCIPL thickness), according to the regression analyses. Therefore, it is possible that decreased macular VD was associated with the dysfunction of RGC, before actual loss of the cell. However, the causal relationship between decreased macular VD and RGC dysfunction, represented by N95 amplitude, remains controversial and should be evaluated further.

Recently, two studies were presented evaluating the relationship between PERG and OCTA in glaucoma patients, from the same group. The prior study assessed the correlations between various parameters, including PERG, OCTA, OCT and VF, in an open angle glaucoma (OAG) patient [33]. According to TJ Lee et al., the N95 amplitude of OAG patients was correlated with decreased macular VD from OCTA, GCIPL thickness, and MD from VF tests. This is consistent with our results. However, the correlation between macular VD and RNFL thickness was not consistent with our study. We thought that this disagreement might be due to the different study subjects. Only NTG patients were included in our study, but the study of TJ Lee et al. included both normal and high-tension glaucoma patients in the study subjects. The precise pathophysiology of this discordance should be studied in a future study. The following study of SY Lee et al. presented the diagnostic power of PERG and OCTA for NTG [34]. In early glaucomam with MD ≥ −6 dB, the association between N95 amplitude and macular VD was significantly associated in correlation analysis, and it was entirely consistent with our results. We expanded the range of subgroup analysis to mild to moderate glaucoma, with MD ≥ −12 dB, and through regression analyses, we presented that macular VD and N95 amplitude could have an effect on each other. Considering the limited use of PERG and rapidly increasing use of OCTA in the clinical setting, OCTA could be used as a functional parameter that could assist PERG in the near future.

This study has several limitations. First, this study only included NTG patients. NTG has some different clinical characteristics from primary open angle glaucoma, with elevated IOP associated with vascular problems, such as disc hemorrhage or systemic vascular diseases [35,36,37]. Therefore, it is difficult to generalize the results of our study to all types of glaucoma. Second, the number of patients with moderate or severe glaucoma was relatively smaller than that of patients with mild glaucoma. Because we focused on the value as a functional marker of macular VD from early glaucoma, the small number of advanced disease cases might not interfere with our conclusion. However, future studies will require a larger sample of patients with advanced disease. Third, the amount of glaucoma medication could have the possibility of being a confounding variable. In our study, the amount of glaucoma medication did not show a significant effect on N95 amplitude or VD. However, mild glaucoma patients, who accounted for a large portion of our study, all used a single glaucoma medication. We thought that this could have the possibility of confounding the results, and it is still necessary to study patients with a greater severity of glaucoma in the future. Finally, the projection artifacts of OCTA could be a limitation of vascular status evaluation. However, the VD measurements of our study were restricted to the superficial retinal layer, including RNFL and GCL, so projection artifacts did not affect the most anterior part of retinal vasculature in the current study.

In conclusion, macular VD was associated with the N95 amplitude of PERG in NTG patients. Macular VD could reflect the dysfunction of RGC, presented by an electrophysiological test in early glaucoma. Macular VD could be used as a surrogate of visual function in patients with early glaucoma.

## Figures and Tables

**Table 1:** Baseline characteristics of study subjects (*n* = 76).

Age (years)	57.47 (±12.78)
Axial length (mm)	25.26 (±1.70)
Male:Female	33:45
Hypertension	21 (27.63%)
Diabetes	6 (7.89%)
Systemic vascular dysregulation *	14 (18.42%)
Disc hemorrhage	5 (6.58%)
Intraocular pressure (mmHg)	14.07 (±2.63)
SITA 24-2 MD (dB)	−5.70 (±6.27)
SITA 24-2 PSD (dB)	5.44 (±4.25)
Average RNFL thickness (μm)	73.16 (±11.22)
Average GCIPL thickness (μm)	66.79 (±9.38)
Pattern ERG	
P50 latency (ms)	50.41 (±3.53)
N95 latency (ms)	102.42 (±7.34)
P50 amplitude (μV)	2.83 (±0.92)
N95 amplitude (μV)	4.92 (±1.75)
Macular VD (%)	
Superior	47.91 (±5.29)
Nasal	42.34 (±5.96)
Inferior	47.48 (±5.67)
Temporal	43.73 (±4.23)
FAZ	14.71 (±5.36)
Average (excluding FAZ) ^†^	45.36 (±3.39)

MD: mean deviation; PSD: pattern standard deviation; RNFL: retinal nerve fiber layer; GCIPL: ganglion cell layer and innter plexiform layer; ERG: electroretinogram; VD: vessel density; FAZ: foveal avascular zone; * Systemic vascular dysregulation included history of migraine, cold extremities, cerebrovascular problems, angina or arrhythmia; ^†^ Average macular VD was calculated by the mean value of VD from superior, nasal, inferior and temporal area.

**Table 2:** Comparisons of functional and structural parameters between higher and lower groups of average macular VD ^†^ in mild to moderate glaucoma (MD ≥ −12 dB).

	Higher VD (*n* = 36)	Lower VD (*n* = 28)	*p* Value
Average RNFL thickness (μm)	73.92 (±9.74)	77.75 (±10.22)	0.132
Average GCIPL thickness (μm)	68.97 (±6.97)	68.14 (±10.07)	0.711
SITA 24-2 MD (dB)	−2.99 (±3.00)	−4.23 (±3.12)	0.346
SITA 24-2 PSD (dB)	3.92 (±3.17)	4.52 (±3.21)	0.632
Pattern ERG			
P50 latency (ms)	50.35 (±3.31)	50.91 (±3.49)	0.514
N95 latency (ms)	103.60 (±7.71)	101.19 (±7.18)	0.206
P50 amplitude (μV)	2.95 (±1.01)	2.75 (±0.93)	0.415
N95 amplitude (μV)	5.52 (±1.88)	4.65 (±1.56)	0.048 *

VD: vessel density; RNFL: retinal nerve fiber layer; GCIPL: ganglion cell layer and innter plexiform layer; MD: mean deviation; PSD: pattern standard deviation; ERG: electroretinogram; Student’s *t* tests were used; ^†^ Average macular VD was calculated by the mean value of VD from superior, nasal, inferior and temporal area; Asterisk (*) indicates statistically significant values, with *p* < 0.05.

**Table 3:** Factors associated with the average macular VD in total subjects.

	Univariate		Multivariate	
	ß ± SE	*p* Value	ß ± SE	*p* Value
Average RNFL thickness	0.019 ± 0.035	0.598		
Average GCIPL thickness	0.085 ± 0.040	0.038 *	0.013 ± 0.049	0.798
SITA 24-2 MD	0.202 ± 0.058	0.001 *	0.170 ± 0.088	0.057
SITA 24-2 PSD	−0.216 ± 0.089	0.018 *	0.131 ± 0.128	0.308
Pattern ERG				
P50 latency	−0.094 ± 0.111	0.402		
N95 latency	0.016 ± 0.054	0.77		
P50 amplitude	0.696 ± 0.420	0.102		
N95 amplitude	0.683 ± 0.211	0.002 *	0.498 ± 0.231	0.035 *

VD: vessel density; RNFL: retinal nerve fiber layer; GCIPL: ganglion cell layer and innter plexiform layer; MD: mean deviation; PSD: pattern standard deviation; ERG: electroretinogram; Variables with *p* values < 0.1 on univariate analysis were included in the multivariate analysis; Asterisk (*) indicates statistically significant values, with *p* < 0.05.

**Table 4:** Factors associated with the average macular VD in mild to moderate glaucoma (MD ≥ −12 dB) (*n* = 64).

	Univariate		Multivariate Model 1		Multivariate Model 2		Multivariate Model 3	
	ß ± SE	*p* Value	ß ± SE	*p* Value	ß ± SE	*p* Value	ß ± SE	*p* Value
Average RNFL thickness	−0.040 ± 0.041	0.336					−0.091 ± 0.047	0.058
Average GCIPL thickness	0.063 ± 0.050	0.213					0.086 ± 0.057	0.138
SITA 24-2 MD	0.212 ± 0.133	0.117	0.171 ± 0.136	0.212	0.139 ± 0.133	0.301		
SITA 24-2 PSD	−0.120 ± 0.132	0.364						
Pattern ERG								
P50 latency	−0.149 ± 0.123	0.231						
N95 latency	0.035 ± 0.056	0.528						
P50 amplitude	0.702 ± 0.424	0.103	0.580 ± 0.433	0.185				
N95 amplitude	0.568 ± 0.224	0.014 *			0.509 ± 0.231	0.032 *	0.510 ± 0.234	0.033 *

VD: vessel density; RNFL: retinal nerve fiber layer; GCIPL: ganglion cell layer and inner plexiform layer; MD: mean deviation; PSD: pattern standard deviation; ERG: electroretinogram; Variables with *p* values < 0.2 on univariate analysis were included and enter mode was used in the multivariate analysis model 1 and 2. P 50 amplitude and N95 amplitude had strong positive correlation (r = 0.694, *p* < 0.001), so, model 1 and 2 included only one variable of two, respectively. Backward elimination model was used in multivariate analysis model 3. Asterisk (*) indicates statistically significant values, with *p* < 0.05.

**Table 5:** Factors associated with the N95 amplitude in total subjects.

	Univariate		Multivariate	
	ß ± SE	*p* Value	ß ± SE	*p* Value
Average RNFL thickness	0.039 ± 0.018	0.030 *	0.019 ± 0.022	0.383
Average GCIPL thickness	0.076 ± 0.021	0.001 *	0.049 ± 0.027	0.074
Macular VD				
Superior	0.133 ± 0.035	<0.001 *		
Nasal	0.022 ± 0.034	0.521		
Inferior	0.058 ± 0.035	0.106		
Temporal	0.113 ± 0.046	0.017 *		
FAZ	−0.045 ± 0.038	0.230		
Average (excluding FAZ) ^†^	0.181 ± 0.056	0.002 *	0.148 ± 0.061	0.019 *

RNFL: retinal nerve fiber layer; GCIPL: ganglion cell layer and inner plexiform layer; VD: vessel density; Variables with *p* values < 0.1 on univariate analysis were included in the multivariate analysis; Average macular VD and superior or temporal VD had strong positive correlation (r = 0.627 and 0.704, respectively; both *p* < 0.001), so, only average macular VD was included in the multivariate analysis; ^†^ Average macular VD was calculated by the mean value of VD from superior, nasal, inferior and temporal area. Asterisk (*) indicates statistically significant values, with *p* < 0.05.

**Table 6:** Factors associated with the N95 amplitude in mild to moderate glaucoma (MD ≥ −12 dB) (*n* = 64).

	Univariate		Multivariate	
	ß ± SE	*p* Value	ß ± SE	*p* Value
Average RNFL thickness	0.020 ± 0.022	0.377		
Average GCIPL thickness	0.064 ± 0.027	0.020 *	0.055 ± 0.026	0.042 *
Macular VD				
Superior	0.137 ± 0.041	0.001 *		
Nasal	0.032 ± 0.036	0.376		
Inferior	0.020 ± 0.044	0.644		
Temporal	0.105 ± 0.052	0.046 *		
FAZ	−0.017 ± 0.044	0.697		
Average (excluding FAZ) ^†^	0.165 ± 0.065	0.014 *	0.143 ± 0.068	0.040 *

RNFL: retinal nerve fiber layer; GCIPL: ganglion cell layer and inner plexiform layer; VD: vessel density; Variables with *p* values < 0.1 on univariate analysis were included in the multivariate analysis; Average macular VD and superior or temporal VD had strong positive correlation (r = 0.723 and 0.685, respectively; both *p* < 0.001), so, only average macular VD was included in the multivariate analysis; ^†^ Average macular VD was calculated by the mean value of VD from superior, nasal, inferior and temporal area; Asterisk (*) indicates statistically significant values, with *p* < 0.05.

## Data Availability

The datasets generated and analyzed during the current study are available from the corresponding author on reasonable request.

## References

[1] Foster P.J., Buhrmann R., Quigley H.A., Johnson G.J. (2002). The definition and classification of glaucoma in prevalence surveys. Br. J. Ophthalmol..

[2] Wu Z., Medeiros F.A. (2018). Recent developments in visual field testing for glaucoma. Curr. Opin. Ophthalmol..

[3] Medeiros F.A., Zangwill L.M., Bowd C., Mansouri K., Weinreb R.N. (2012). The structure and function relationship in glaucoma: Implications for detection of progression and measurement of rates of change. Investig. Ophthalmol. Vis. Sci..

[4] Jung K.I., Park C.K. (2017). Detection of Functional Change in Preperimetric and Perimetric Glaucoma Using 10-2 Matrix Perimetry. Am. J. Ophthalmol..

[5] Ventura L.M., Sorokac N., De Los Santos R., Feuer W.J., Porciatti V. (2006). The relationship between retinal ganglion cell function and retinal nerve fiber thickness in early glaucoma. Investig. Ophthalmol. Vis. Sci..

[6] Kerrigan-Baumrind L.A., Quigley H.A., Pease M.E., Kerrigan D.F., Mitchell R.S. (2000). Number of ganglion cells in glaucoma eyes compared with threshold visual field tests in the same persons. Investig. Ophthalmol. Vis. Sci..

[7] Chew S.S., Kerr N.M., Wong A.B., Craig J.P., Chou C.Y., Danesh-Meyer H.V. (2016). Anxiety in visual field testing. Br. J. Ophthalmol..

[8] Glen F.C., Baker H., Crabb D.P. (2014). A qualitative investigation into patients’ views on visual field testing for glaucoma monitoring. BMJ Open.

[9] Phu J., Khuu S.K., Yapp M., Assaad N., Hennessy M.P., Kalloniatis M. (2017). The value of visual field testing in the era of advanced imaging: Clinical and psychophysical perspectives. Clin. Exp. Optom..

[10] Berninger T.A., Arden G.B. (1988). The pattern electroretinogram. Eye.

[11] Bach M., Poloschek C.M. (2013). Electrophysiology and glaucoma: Current status and future challenges. Cell Tissue Res..

[12] Bach M., Unsoeld A.S., Philippin H., Staubach F., Maier P., Walter H.S., Bomer T.G., Funk J. (2006). Pattern ERG as an early glaucoma indicator in ocular hypertension: A long-term, prospective study. Investig. Ophthalmol. Vis. Sci.

[13] Wilsey L.J., Fortune B. (2016). Electroretinography in glaucoma diagnosis. Curr. Opin. Ophthalmol..

[14] Jafarzadehpour E., Radinmehr F., Pakravan M., Mirzajani A., Yazdani S. (2013). Pattern electroretinography in glaucoma suspects and early primary open angle glaucoma. J. Ophthalm. Vis. Res..

[15] Jung K.I., Jeon S., Shin D.Y., Lee J., Park C.K. (2020). Pattern Electroretinograms in Preperimetric and Perimetric Glaucoma. Am. J. Ophthalmol..

[16] Jeon S.J., Park H.L., Jung K.I., Park C.K. (2019). Relationship between pattern electroretinogram and optic disc morphology in glaucoma. PLoS ONE.

[17] Yarmohammadi A., Zangwill L.M., Diniz-Filho A., Suh M.H., Manalastas P.I., Fatehee N., Yousefi S., Belghith A., Saunders L.J., Medeiros F.A. (2016). Optical Coherence Tomography Angiography Vessel Density in Healthy, Glaucoma Suspect, and Glaucoma Eyes. Investig. Ophthalmol. Vis. Sci..

[18] Wang X., Jiang C., Ko T., Kong X., Yu X., Min W., Shi G., Sun X. (2015). Correlation between optic disc perfusion and glaucomatous severity in patients with open-angle glaucoma: An optical coherence tomography angiography study. Graefes Arch. Clin. Exp. Ophthalmol..

[19] Wu J., Sebastian R.T., Chu C.J., McGregor F., Dick A.D., Liu L. (2019). Reduced Macular Vessel Density and Capillary Perfusion in Glaucoma Detected Using OCT Angiography. Curr. Eye Res..

[20] Yarmohammadi A., Zangwill L.M., Diniz-Filho A., Suh M.H., Yousefi S., Saunders L.J., Belghith A., Manalastas P.I., Medeiros F.A., Weinreb R.N. (2016). Relationship between Optical Coherence Tomography Angiography Vessel Density and Severity of Visual Field Loss in Glaucoma. Ophthalmology.

[21] Mills R.P., Budenz D.L., Lee P.P., Noecker R.J., Walt J.G., Siegartel L.R., Evans S.J., Doyle J.J. (2006). Categorizing the stage of glaucoma from pre-diagnosis to end-stage disease. Am. J. Ophthalmol..

[22] Fernandez-Vigo J.I., Kudsieh B., Shi H., Arriola-Villalobos P., Donate-Lopez J., Garcia-Feijoo J., Ruiz-Moreno J.M., Fernandez-Vigo J.A. (2020). Normative database and determinants of macular vessel density measured by optical coherence tomography angiography. Clin. Exp. Ophthalmol..

[23] Medeiros F.A., Alencar L.M., Zangwill L.M., Bowd C., Sample P.A., Weinreb R.N. (2009). Prediction of functional loss in glaucoma from progressive optic disc damage. Arch. Ophthalmol..

[24] Harwerth R.S., Carter-Dawson L., Smith E.L., Barnes G., Holt W.F., Crawford M.L. (2004). Neural losses correlated with visual losses in clinical perimetry. Investig. Ophthalmol. Vis. Sci..

[25] Hood D.C., Kardon R.H. (2007). A framework for comparing structural and functional measures of glaucomatous damage. Prog. Retin. Eye Res..

[26] Ventura L.M., Porciatti V. (2005). Restoration of retinal ganglion cell function in early glaucoma after intraocular pressure reduction: A pilot study. Ophthalmology.

[27] Yarmohammadi A., Zangwill L.M., Diniz-Filho A., Saunders L.J., Suh M.H., Wu Z., Manalastas P.I.C., Akagi T., Medeiros F.A., Weinreb R.N. (2017). Peripapillary and Macular Vessel Density in Patients with Glaucoma and Single-Hemifield Visual Field Defect. Ophthalmology.

[28] Gaier E.D., Wang M., Gilbert A.L., Rizzo J.F., Cestari D.M., Miller J.B. (2018). Quantitative analysis of optical coherence tomographic angiography (OCT-A) in patients with non-arteritic anterior ischemic optic neuropathy (NAION) corresponds to visual function. PLoS ONE.

[29] Shoji T., Zangwill L.M., Akagi T., Saunders L.J., Yarmohammadi A., Manalastas P.I.C., Penteado R.C., Weinreb R.N. (2017). Progressive Macula Vessel Density Loss in Primary Open-Angle Glaucoma: A Longitudinal Study. Am. J. Ophthalmol..

[30] Hou H., Moghimi S., Proudfoot J.A., Ghahari E., Penteado R.C., Bowd C., Yang D., Weinreb R.N. (2020). Ganglion Cell Complex Thickness and Macular Vessel Density Loss in Primary Open-Angle Glaucoma. Ophthalmology.

[31] Baltan S., Inman D.M., Danilov C.A., Morrison R.S., Calkins D.J., Horner P.J. (2010). Metabolic vulnerability disposes retinal ganglion cell axons to dysfunction in a model of glaucomatous degeneration. J. Neurosci..

[32] Swanson R.A., Sagar S.M., Sharp F.R. (1989). Regional brain glycogen stores and metabolism during complete global ischaemia. Neurol. Res..

[33] Lee T., Seo D.R., Kim J.Y., Choi W., Lee S.Y., Lee J.M., Seong G.J., Kim C.Y., Bae H.W. (2020). Relationship between N95 Amplitude of Pattern Electroretinogram and Optical Coherence Tomography Angiography in Open-Angle Glaucoma. J. Clin. Med..

[34] Lee S.Y., Son N.H., Bae H.W., Seong G.J., Kim C.Y. (2021). The role of pattern electroretinograms and optical coherence tomography angiography in the diagnosis of normal-tension glaucoma. Sci. Rep..

[35] Barry C.J., Cooper R.L., Eikelboom R.H. (1997). Optic disc haemorrhages and vascular abnormalities in a glaucoma population. Aust. N. Z. J. Ophthalmol..

[36] Leung D.Y., Tham C.C., Li F.C., Kwong Y.Y., Chi S.C., Lam D.S. (2009). Silent cerebral infarct and visual field progression in newly diagnosed normal-tension glaucoma: A cohort study. Ophthalmology.

[37] Suzuki J., Tomidokoro A., Araie M., Tomita G., Yamagami J., Okubo T., Masumoto T. (2004). Visual field damage in normal-tension glaucoma patients with or without ischemic changes in cerebral magnetic resonance imaging. Jpn. J. Ophthalmol..

